# SIRT1 prevents cigarette smoking-induced lung fibroblasts activation by regulating mitochondrial oxidative stress and lipid metabolism

**DOI:** 10.1186/s12967-022-03408-5

**Published:** 2022-05-14

**Authors:** Yue Zhang, Ting Li, Miaoxia Pan, Wei Wang, Wenhui Huang, Yafei Yuan, Zhanzhan Xie, Yixin Chen, Jun Peng, Xu Li, Ying Meng

**Affiliations:** 1 Department of Respiratory and Critical Care Medicine, Nanfang Hospital, Southern Medical University, Guangzhou, Guangdong China; 2 Department of Emergency Medicine, Nanfang Hospital, Southern Medical University, Guangzhou, Guangdong China; 3 Ministry of Education, Key Laboratory of Hainan Trauma and Disaster Rescue, College of Emergency and Trauma, Hainan Medical University, Haikou, China

**Keywords:** Cigarette smoking, SIRT1, Mitochondrial oxidative stress, Autophagy, Lipid metabolism

## Abstract

**Background:**

Cigarette smoking (CS) is a strong risk factor for idiopathic pulmonary fibrosis (IPF). It can activate lung fibroblasts (LF) by inducing redox imbalance. We previously showed that clearing mitochondrial reactive oxygen species (mtROS) protects against CS-induced pulmonary fibrosis. However, the precise mechanisms of mtROS in LF need further investigation. Here we focused on mtROS to elucidate how it was regulated by CS in LF and how it contributed to LF activation.

**Methods:**

We treated cells with 1% cigarette smoking extract (CSE) and examined mtROS level by MitoSOX^™^ indicator. And the effect of CSE on expression of SIRT1, SOD2, mitochondrial NOX4 (mtNOX4), fatty acid oxidation (FAO)-related protein PPARα and CPT1a and LF activation marker Collagen I and α-SMA were detected. Nile Red staining was performed to show cellular lipid content. Then, lipid droplets, autophagosome and lysosome were marked by Bodipy 493/503, LC3 and LAMP1, respectively. And lipophagy was evaluated by the colocalization of lipid droplets with LC3 and LAMP1. The role of autophagy on lipid metabolism and LF activation were explored. Additionally, the effect of mitochondria-targeted ROS scavenger mitoquinone and SIRT1 activator SRT1720 on mitochondrial oxidative stress, autophagy flux, lipid metabolism and LF activation were investigated in vitro and in vivo.

**Results:**

We found that CS promoted mtROS production by increasing mtNOX4 and decreasing SOD2. Next, we proved mtROS inhibited the expression of PPARα and CPT1a. It also reduced lipophagy and upregulated cellular lipid content, suggesting lipid metabolism was disturbed by CS. In addition, we showed both insufficient FAO and lipophagy resulted from blocked autophagy flux caused by mtROS. Moreover, we uncovered decreased SIRT1 was responsible for mitochondrial redox imbalance. Furthermore, we proved that both SRT1720 and mitoquinone counteracted the effect of CS on NOX4, SOD2, PPARα and CPT1a in vivo.

**Conclusions:**

We demonstrated that CS decreased SIRT1 to activate LF through dysregulating lipid metabolism, which was due to increased mtROS and impaired autophagy flux. These events may serve as therapeutic targets for IPF patients.

**Supplementary Information:**

The online version contains supplementary material available at 10.1186/s12967-022-03408-5.

## Introduction

Mitochondrial oxidative stress is a critical player in idiopathic pulmonary fibrosis (IPF) [1] and TGF-β-induced lung fibroblast (LF) activation [2]. Targeting mitochondrial reactive oxygen species (mtROS) can alleviate CS-related lung fibrosis in vivo [3]. Lung fibroblasts (LF) are effector cells in the pathogenesis of IPF. It can be activated by CS [4]. However, whether mtROS of LF was induced by CS and how mtROS activated LF is uncertain. NADPH oxidases (NOXs) is one of the main sources of ROS. In LF isolated from IPF patients, NOX4 is elevated while NOX1, 2 and 5 have no significant change [5]. In addition, NOX4 can be upregulated by CS [4]. Several lines of evidences manifested that NOX4 can be located in mitochondria since it contains a mitochondrial targeting signal [1]. Thus, we aim to investigate whether and how CS increased mitochondrial NOX4 (mtNOX4) and thereby produced more mtROS.

Mitochondria is the powerhouse and center of metabolism in eukaryotic cells. Evidences indicated that mitochondrial oxidative stress is closely associated with metabolic disorders [6]. In fibrotic lungs, fatty acid (FA) and total lipid content [7] are increased. And high fat diet can aggravate experimental pulmonary fibrosis [8], implying lipid metabolism participates in lung fibrosis. In addition, lipid metabolism can be affected by CS [9]. However, whether CS-induced mitochondrial oxidative stress dysregulated lipid metabolism and subsequentially activated LF and its underlying mechanisms are unclear. Fatty acid oxidation (FAO), for which mitochondria is an important place, is a process closely associated with lipid homeostasis. CPT1a is a key rate-limiting enzyme of mitochondrial FAO and is reported to be involved in fibrotic diseases, such as kidney [10] and liver fibrosis [11]. Moreover, its upstream regulator, PPARα, also exhibits anti-fibrotic effect in liver [12], kidney [13], heart [14] and lung [15]. Therefore, its noteworthy to find out whether CS-induced mtROS activated LF through dysregulating PPARα/CPT1a-mediated FAO and lipid metabolism, which may be targets for lung fibrosis therapy.

SIRT1, a lysine deacetylase, is a modulator of mitochondrial oxidative stress [3] and NOX4 expression [16]. It has been reported that its anti-fibrotic effect is associated with decreased mtROS [3, 17]. It can also inhibit fibroblasts activation [3, 17] and modulate lipid metabolism [19]. However, whether SIRT1 protect against LF activation by regulating mtNOX4- related mtROS and lipid metabolism and its mechanisms need to be further investigated.

In the present study, we explored how mitochondrial redox balance was disrupted by CS and how mtROS activated LF. We found imbalance of mtNOX4 and SOD2 resulting from decreased SIRT1 was responsible for CS-induced mtROS. mtROS impaired autophagy flux to activate LF by inhibiting PPARα/CPT1-related FAO and lipophagy.

## Materials and methods

### Animals

Six-week-old male C57 mice were randomly divided into four groups: Control, CS, CS + MitoQ (1.5 mg/kg, HY-100116, MCE), CS + SRT1720 (20 mg/kg, S1129, Selleck). 10 mice in each group. In the three CS groups, mice were placed in an 80 × 35 × 33 cm chamber and exposed to 5 commercial cigarettes for 30 min each time, and two times a day. For MitoQ and SRT1720 group, MitoQ and SRT1720 were injected intraperitoneally into mice every two days or each day, respectively. 4 weeks later, lungs were harvested. All mice were obtained from Southern Medical University Animal Center (Guangzhou, China) and housed in standard environment. All experimental procedures on mice were approved by Committee on the Ethics of Animal Experiments of Southern Medical University (Permit No. SYXK 2015-0056).

### Cell culture and treatment

Primary LF were isolated from 6-week-old mice as previously described [20] and cultured with DMEM containing 15% FBS at 37 °C. Passage 2 cells were treated with MitoQ (50 nM, HY-100116, MCE), fenofibrate (10 μM, T1149, Topscience), oleic acid (10 μM, S4707, Selleck), etomoxir (50 μM, S8244, Selleck), bafilomycin (5 nM, S1413, Selleck) and SRT1720 (4 μM, S1129, Selleck) (Additional file 1). The dosages of these compounds were based on published papers. And MTT test for them was performed (Additional file 2).

### Preparation of cigarette smoke extract (CSE)

Firstly, smoke of 1 cigarette was collected by a 20 ml syringe which contained 2 ml PBS. Then, the absorbance of the solution was detected at the wavelength of 490 nm. The concentration was considered as 100% when the absorbance was 0.1. Next, adjusted its pH to 7.4 and filtered it with 0.2 μm membrane. The obtained CSE was kept in 4 °C and applied within 20 min.

### MitoSOX red, lysotracker red, nile red and BODIPY 493/503 staining

Living cells were incubated with MitoSOX^™^ Red (2.5 μM, M36008, Invitrogen), Lysotracker Red DND-99 (50 nM, L7528, Invitrogen), Nile Red (1 μM, HY-D0718, MCE) or BODIPY staining solution (2 μM, GC42959, GLPBIO) for 15 min at 37 °C in dark. Then, cells were washed with HBSS/Ca/Mg and analyzed by fluorescence microscopy (IX73, Olympus or Imager D2, Carl Zeiss).

### Immunofluorescence staining

Lung sections or cells treated with 4% paraformaldehyde for 15 min and 0.2% triton for 10 min were blocked with 5% goat serum for 60 min at room temperature. Then, they were incubated with primary antibodies at 4 °C for overnight and stained with FITC- (A0562, beyotime) and Coralite594-conjugated secondary antibody (SA00013-4, Proteintech) at room temperature for 1 h, after which nuclear were stained with DAPI (F6057, sigma). Pictures were captured with confocal microscopy (LSM880, Carl Zeiss) or fluorescence microscopy (Imager D2, Carl Zeiss). Primary antibodies used here were as follows: anti-NOX4 (ab154244, abcam), anti-COX IV (200147, ZENBIO), anti-LC3 II/I (A5179, bimake), anti-SOD2 (A5377, bimake), anti-collagen I (ABM40379, Abbkine), anti-CPT1a (15184-1-AP, proteintech) and anti-PPARα (Abp55667, Abbkine).

### Western blot analysis

The relative expression of total protein or mitochondrial protein were detected by western blot. Antibodies used here were as follows: Collagen I (ab260043, abcam), α-SMA (ab7817, abcam), VDAC1 (A5224, Bimake), p62 (18420-1-AP, proteintech), LC3 II/I (A5179, bimake), SIRT1 (13161-1-AP, Proteintech), GAPDH (RM2001; Ray Antibody Biotech), and secondary antibodies (92632210, 92632211, Licor). The bands were visualized by Odyssey System (LI-COR).

### Statistical analysis

Results were shown as mean ± SD. Data analysis were performed by SPSS 22.0 (SPSS Inc., Chicago, IL, USA). Intergroup comparison of the mean values was analyzed by one-way analysis of variance (ANOVA). Statistical significance was defined as p < 0.05.

## Results

### Cigarette smoke extract (CSE) increased mtNOX4/SOD2-mediated mtROS to activate primary LF

**Fig. 1:**
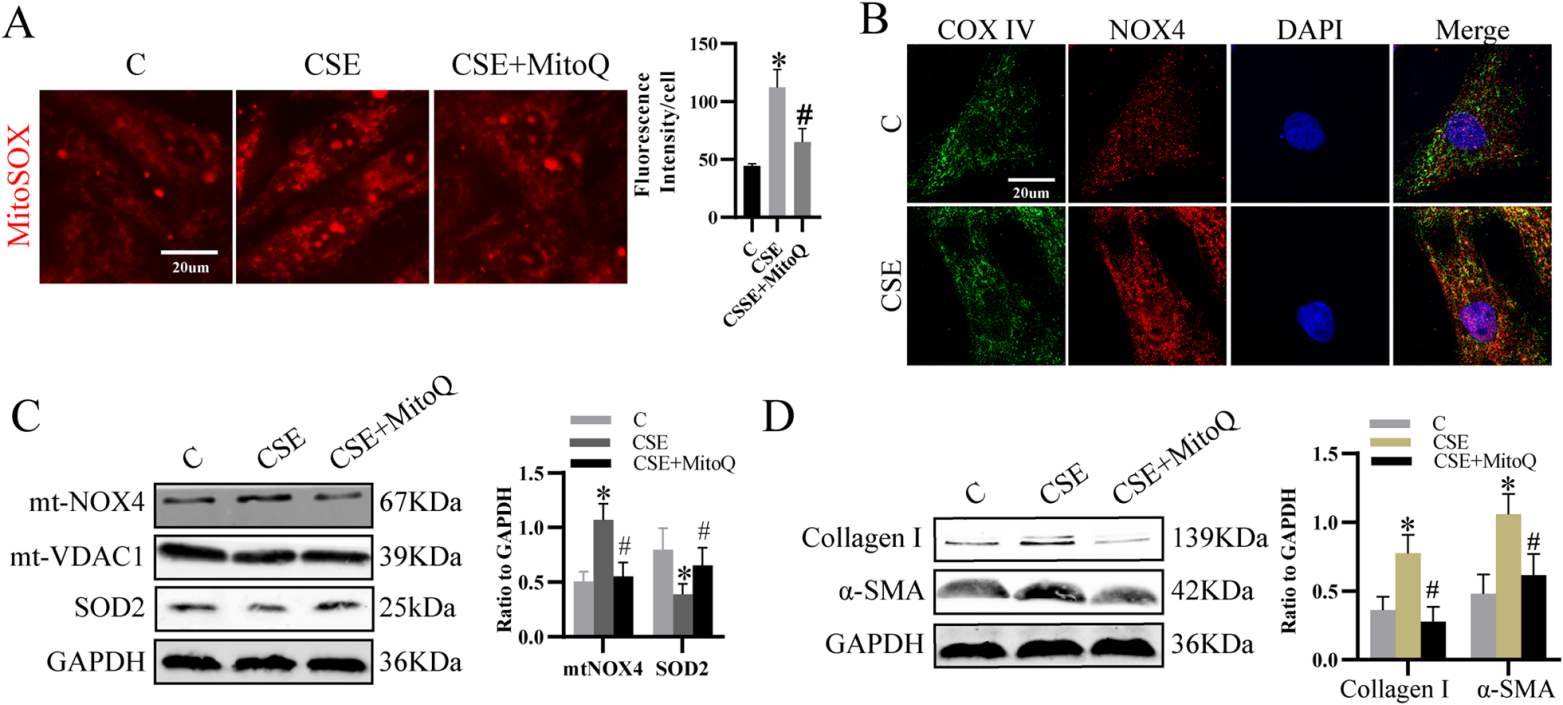
CSE activated LF by inducing mtNOX4- and SOD2- mediated mitochondrial oxidative stress. **A** Cells pretreated with or without MitoQ for 1 h were treated with CSE for 24 h, and then incubated with MitoSOX Red indicator for 10 min in dark. Then, pictures were captured by fluorescence microscopy. **B** Co-localization of NOX4 and COX IV was examined by immunofluorescence. **C** Protein levels of SOD2 in total cell lysate and NOX4 in mitochondrial lysate were detected. **D** Cells pretreated with or without MitoQ for 1 h were stimulated with CSE for 24 h. Then, the level of Collagen I and α-SMA were examined. *p < 0.05 versus control, ^#^*p* < 0.05 versus CSE (n = 3). *CSE* cigarette smoke extract, *mtNOX4* mitochondrial NADPH oxidase 4, *MitoQ* mitoquinone

To make clear the effect of CSE on mtROS, we incubated LF with MitoSOX Red, a mitochondria specific superoxide indicator. Results showed that mtROS was promoted by CSE (Fig. 1A). The colocalization of NOX4 with COX IV, a mitochondrial marker, was upregulated (Fig. 1B). The protein level of NOX4 in mitochondrial lysate was also elevated, indicating CSE increased mtNOX4 (Fig. 1C). Since oxidative stress is a result of the imbalance of ROS production and cellular antioxidant defense, we detected the expression of SOD2, the main antioxidant in mitochondria. And we found SOD2 was decreased (Fig. 1C), suggesting mitochondrial redox balance was disturbed. Furthermore, results showed mitochondria-targeted antioxidant mitoquinone (MitoQ) inhibited the expression of collagen I and α-SMA (Fig. 1D), two markers of LF activation, coupled with decreased mtROS and mtNOX4 level and increased SOD2 expression (Fig. 1A, C). Therefore, CSE activated LF by increasing mtROS which may be due to the imbalance of mtNOX4 and SOD2.

### mtROS activated LF by decreasing PPARα/CPT1a-mediated fatty acid oxidation (FAO)

**Fig. 2:**
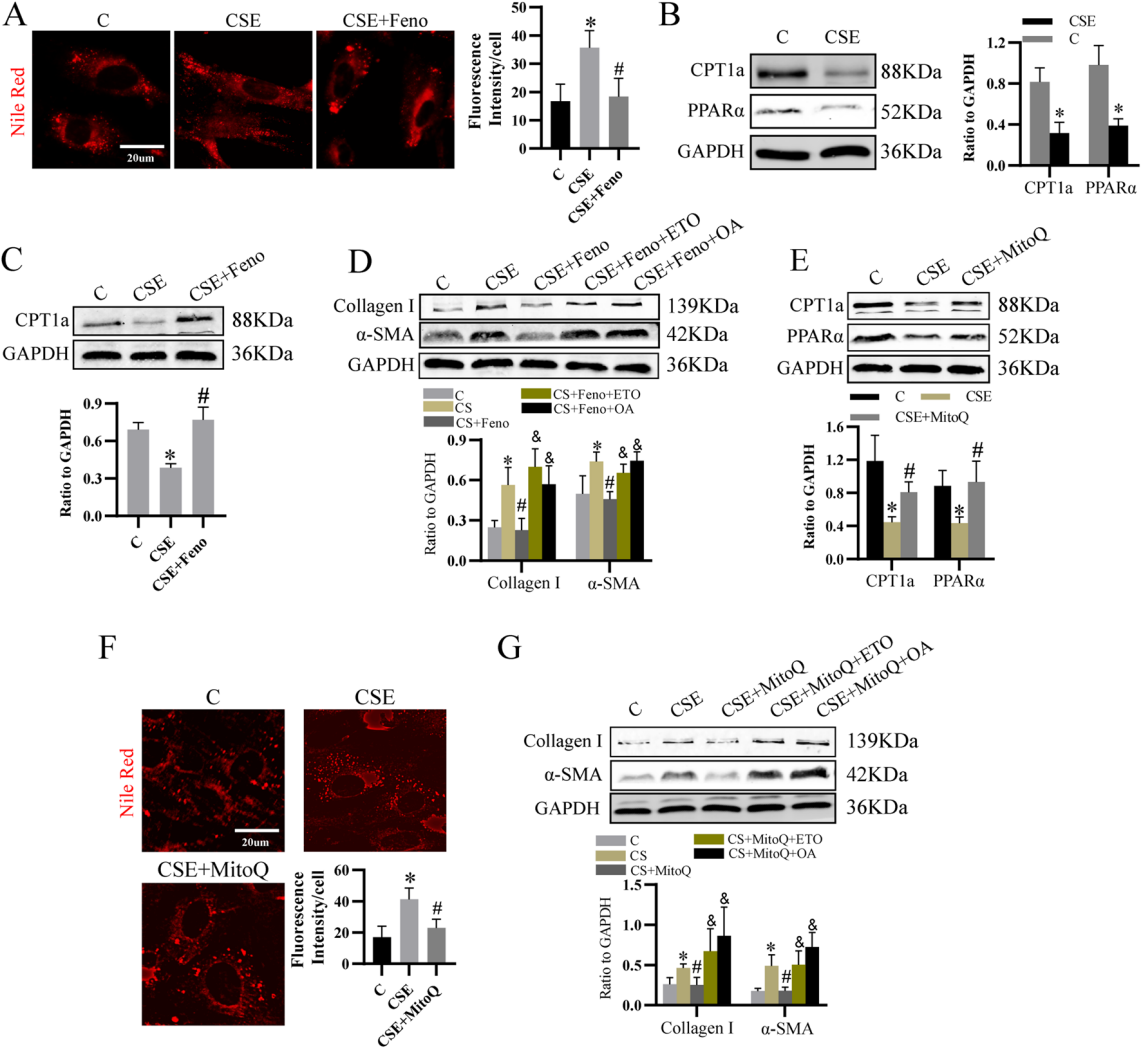
CSE-induced mitochondrial oxidative stress dysregulated lipid metabolism to activate LF. **A** Nile Red staining was performed to show lipid content in cells treated as indicated. **B** The effect of CSE on protein level of CPT1a and PPARα which were related with FAO. **C** The effect of PPARα activator Feno on CPT1a level. **D** Western blot to determine the level of Collagen I and α-SMA. **E** The role of MitoQ in CPT1a and PPARα expression. **F** Nile Red staining. **G** Western blot analysis for Collagen I and α-SMA expression. *p < 0.05 versus control, ^#^*p* < 0.05 versus CSE, ^&^*p* < 0.05 versus CSE + Feno or CSE + MitoQ. *Feno* fenofibrate, *ETO* etomoxir, *OA* oleic acid

Next, we found lipid deposition was increased as indicated by Nile Red staining (Fig. 2A), while CPT1a and PPARα expression was downregulated (Fig. 2B), indicating lipid metabolism was altered by CSE. Then, results showed PPARα activator fenofibrate (Feno) elevated CPT1a level (Fig. 2C) and decreased lipid deposition (Fig. 2A). Furthermore, we found it downregulated the expression of CSE-induced collagen I and α-SMA (Fig. 2D). And both the etomoxir (ETO), a selective inhibitor of CPT1a, and extracellular oleic acid (OA), a fatty acid that was upregulated in fibrotic lungs [21], erased the effect of Feno on collagen I and α-SMA (Fig. 2D). Therefore, CSE decreased PPARα/CPT1a-mediated FAO, which resulted in increased lipid deposition to activate LF.

Then, we examined the effect of mtROS on PPARα/CPT1a-mediated lipid metabolism. Results showed MitoQ increased the level of PPARα and CPT1a and declined lipid deposition (Fig. 2E, F). Moreover, both ETO and OA eliminated the inhibitory effect of MitoQ on collagen I and α-SMA (Fig. 2G), suggesting CSE-induced mtROS impaired PPARα/CPT1a-mediated lipid metabolism to activate LF.

### mtROS dysregulated lipid metabolism by impairing autophagy flux

**Fig. 3:**
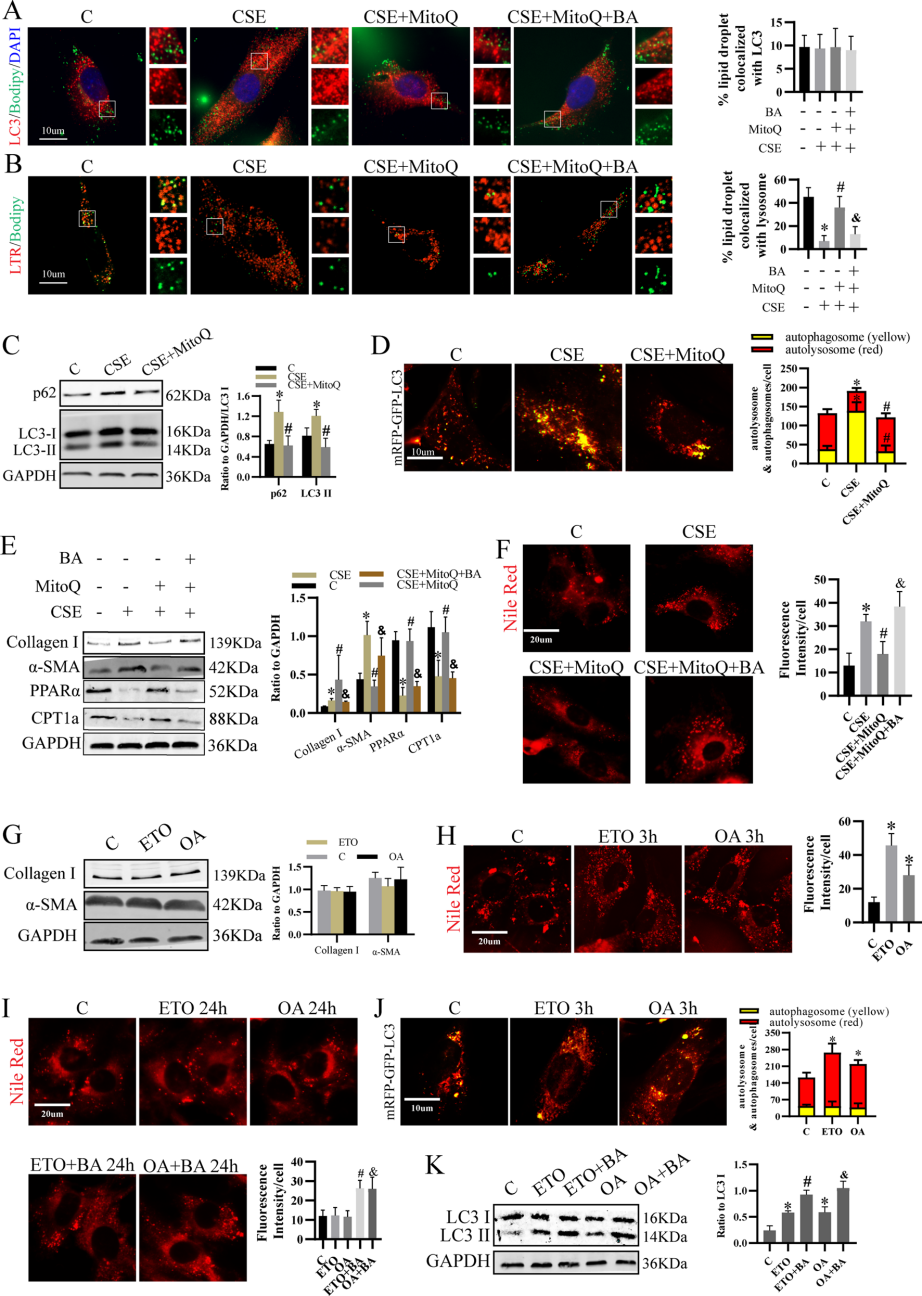
mtROS disturbed lipid metabolism by impaired autophagy flux, which decreased PPARα/CPT1a and lipophagy. **A**, **B** Lipophagy was shown as the co-localization of LC3 (red) (**A**) or LTR (red) (**B**) with LDs indicated by Bodipy 493/503 (green). **C** Protein level of p62, LC3 I and LC3 II. **D** Cells transfected with mRFP-GFP-LC3 adenovirus were treated with CES and MitoQ for 24 h. Then, pictures were captured. Yellow dots were autophagosomes and red were autolysosomes. **E** Protein level of collagen I, α-SMA, PPARα and CPT1a in each group. **F** Lipid content was assessed by Nile Red staining. *p < 0.05 versus control, ^#^*p* < 0.05 versus CSE, ^&^*p* < 0.05 versus CSE + MitoQ (n = 3). **G** Protein level of collagen I and α-SMA of cells treated with ETO or OA alone for 24 h. **H** Nile Red staining was performed to examine lipid content of cells stimulated with ETO or OA for 3 h. **I** Cells pretreated with or without BA for 1 h were treated with ETO or OA for 24 h. Then lipid content was evaluated by Nile Red staining. **J** Autophagosomes (yellow) and autolysosomes (red) were observed. **K** Cells pretreated with or without BA for 1 h were stimulated with ETO or OA for 3 h. Then protein level of LC3 I and LC3 II were detected. *p < 0.05 versus control, ^#^*p* < 0.05 versus ETO, ^&^*p* < 0.05 versus OA (n = 3). *BA* bafilomycin, *LTR* lysotracker red

Lipophagy refers to a process in which the lipid droplets (LDs) are engulfed by autophagosomes and subsequently degraded by lysosomes [22]. It is crucial for lipid homeostasis. We previously revealed that CSE blocked autophagy flux by impairing lysosomes [4]. To find out whether lipophagy was also impaired, LDs were marked by BODIPY 493/503. Results showed that CSE had no effect on the co-localization of LDs with LC3 (Fig. 3A), but decreased the co-localization of LDs with lysosomes, which was stained by Lysotracker Red, an indicator of lysosomes (Fig. 3B), suggesting lipophagy was inhibited by CSE. Next, we explored the regulatory effect of mtROS on lipophagy. We found that MitoQ restored lipophagy (Fig. 3A, B), coupled with improved autophagy flux as indicated by decreased level of LC3 II and p62 and number of autophagosomes and increased number of autolysosomes (Fig. 3C, D). However, in the presence of bafilomycin (BA), a blocker of autophagy flux, lipophagy cannot be induced by MitoQ (Fig. 3A, B), indicating CSE-induced mtROS inhibited lipophagy by disrupting autophagy flux. Not only lipophagy, the effect of MitoQ on PPARα, CPT1a, lipid content, collagen I and α-SMA was also inhibited by BA (Fig. 3E, F), demonstrating CSE-induced mtROS activated LF by impairing autophagy flux which inhibited lipophagy as well as PPARα/CPT1a-mediated FAO.

Interestingly, in the absence of CSE, ETO or OA treatment for 24 h failed to induce LF activation (Fig. 3G). Moreover, lipid content was increased at 3 h while back to baseline level at 24 h (Fig. 3H, I), suggesting LF may have compensatory capacity for lipid metabolism. Then, we explored whether the capacity was associated with autophagy. And we found that after blocking autophagy flux for 24 h by BA, lipid deposition was increased by ETO or OA alone (Fig. 3I). Furthermore, we found the number of autolysosomes were increased at the early time of ETO or OA treatment (Fig. 3J). Consistently, LC3II was upregulated (Fig. 3K). It can be further elevated by BA, which suggested autophagy was activated by ETO or OA in the absence of CSE. Taken together, LF had compensatory capacity for lipid metabolism, which may be modulated by autophagy. These results further confirmed the essential role of autophagy in lipid homeostasis of LF.

### SIRT1 prevented CSE-induced LF activation by regulating lipid metabolism in an autophagy-dependent pathway

**Fig. 4:**
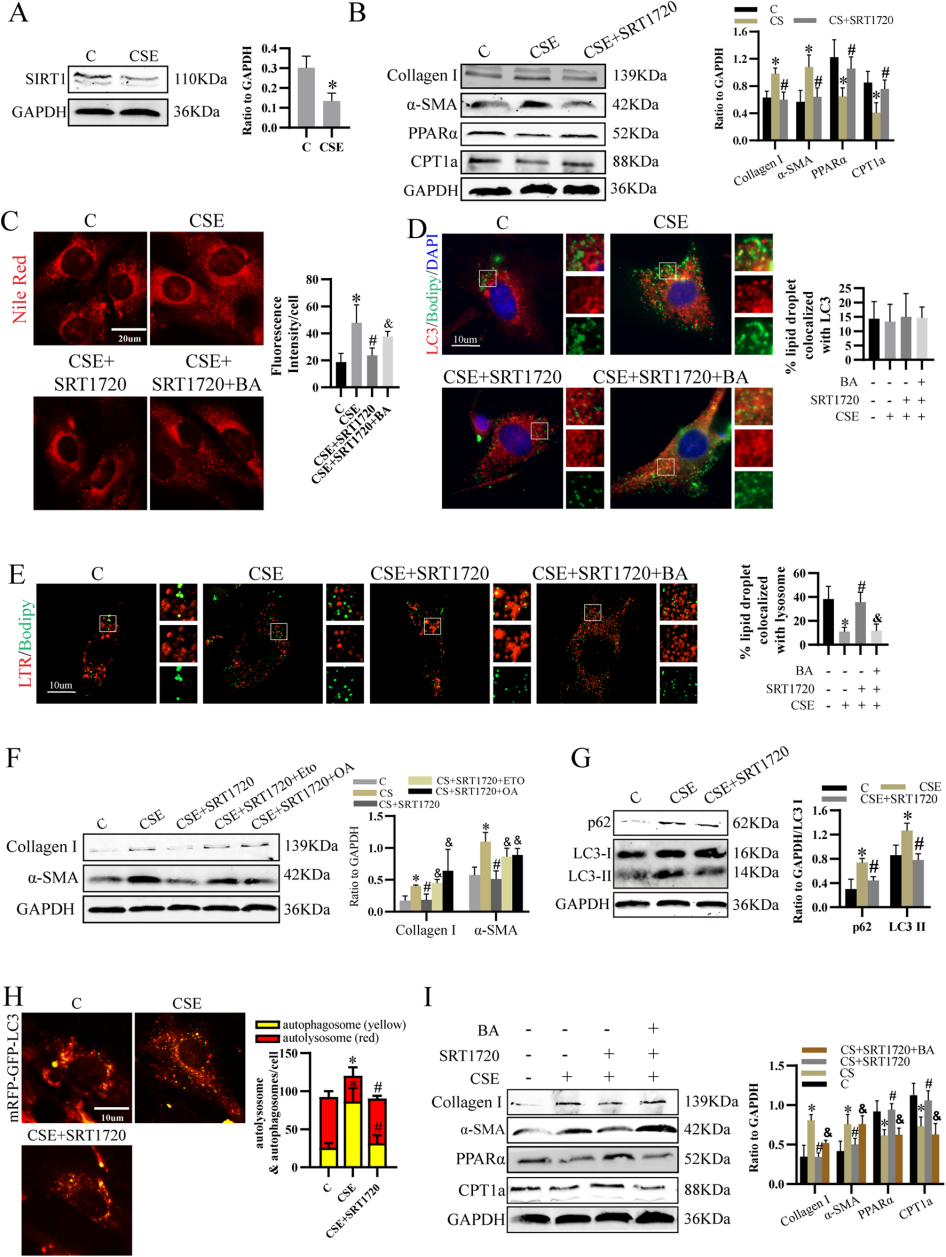
SIRT1 prevented CSE-induced LF activation by regulating lipid metabolism in an autophagy-dependent pathway. **A** Level of SIRT1 was detected. **B** Effect of SIRT1 activator SRT1720 on the expression of Collagen I, α-SMA, CPT1a and PPARα. **C** Nile Red Staining. **D**, **E** Representative pictures of lipophagy as indicated by the co-localization of LC3 (**D**) or LTR (**E**) with Bodipy 493/503. **F**, **G** Western blot analysis for Collagen I, α-SMA, p62, LC3 I and LC3 II. **H** Representative pictures and numbers of autophagosomes (yellow) and autolysosomes (red). **I** Level of collagen I, α-SMA, CPT1a and PPARα were examined. *p < 0.05 versus control, ^#^*p* < 0.05 versus CSE, ^&^*p* < 0.05 versus CSE + SRT1720 (n = 3)

Then, we found SIRT1 expression in LF was decreased by CSE (Fig. 4A). Activating SIRT1 by its activator SRT1720 downregulated the level of collagen I and α-SMA (Fig. 4B), indicating CSE activated LF by inhibiting SIRT1 expression. Next, we explored whether the effect of SIRT1 on LF activation was mediated by lipid metabolism. Results showed SRT1720 increased PPARα and CPT1a level (Fig. 4B) and decreased lipid deposition (Fig. 4C). Although it did not change the colocalization of LDs with LC3 (Fig. 4D), the colocalization of LDs with lysosomes was increased (Fig. 4E). Furthermore, the inhibitory effect of SRT1720 on LF activation was reversed by ETO and OA (Fig. 4F). Thus, CSE dysregulated PPARα/CPT1a and lipophagy by suppressing SIRT1.

Moreover, we found LC3 II and p62 expression (Fig. 4G) and number of autophagosomes were decreased and autolysosomes was increased by SRT1720 (Fig. 4H), which suggested CSE impaired autophagy flux through reducing SIRT1. To make clear whether SIRT1 modulated lipid metabolism and LF activation in an autophagy-dependent pathway, BA were used to pretreat cells. And results showed SRT1720 failed to regulate the expression of collagen I, α-SMA, PPARα and CPT1a (Fig. 4I), lipophagy (Fig. 4D, E) and lipid deposition (Fig. 4C) in BA-pretreated cells. Consequently, these results demonstrated that SIRT1 inhibited CSE-induced LF activation by modulating lipid metabolism in an autophagy-dependent pathway.

### The protective effect of SIRT1 was associated with mitochondrial redox balance

**Fig. 5:**
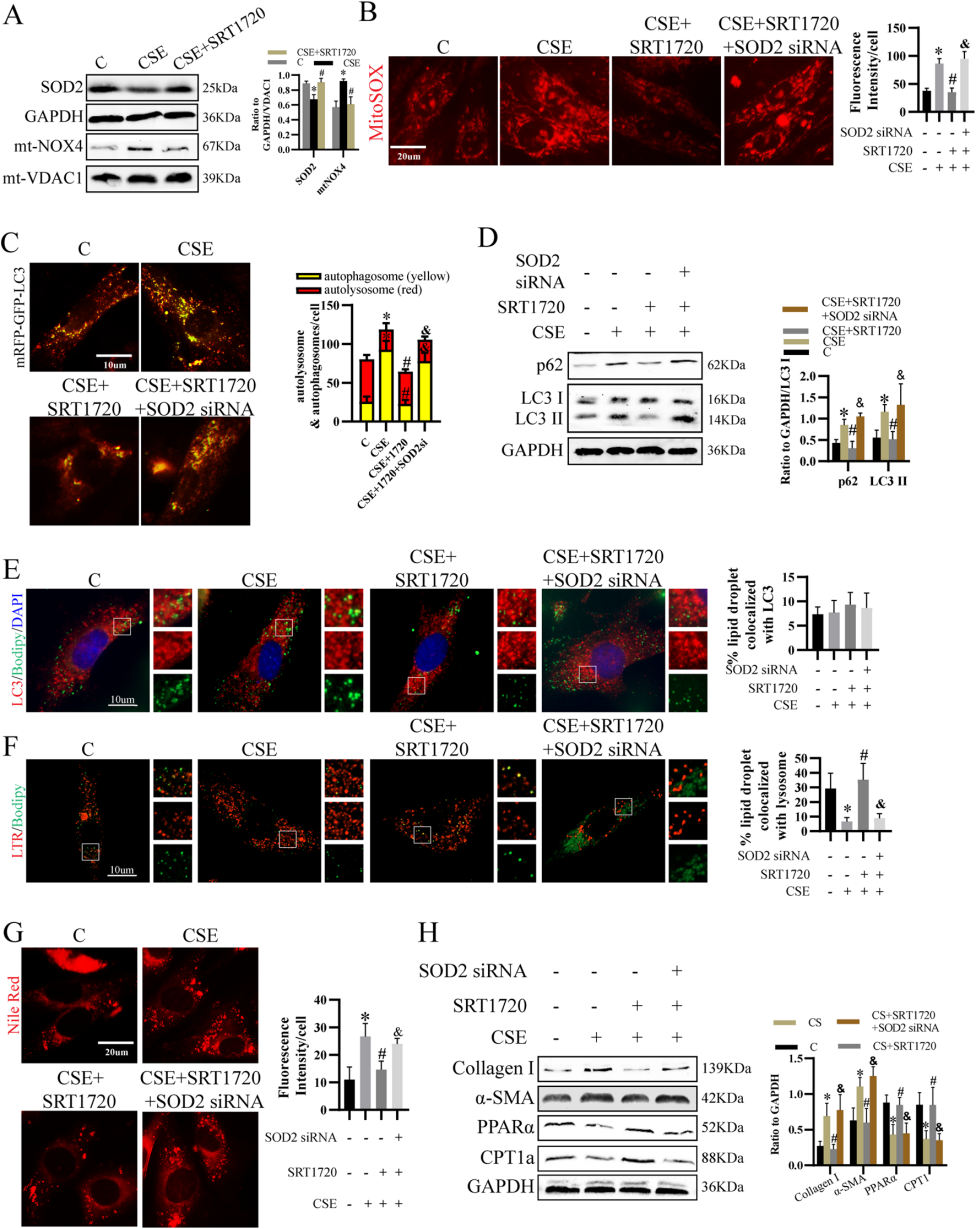
The protective effect of SIRT1 was mediated by mitochondrial redox balance. **A** Level of SOD2 and mtNOX4. **B** mtROS level were examined by mitoSOX indicator. **C** Autophagosomes (yellow) and autolysosomes (red) were evaluated. **D** SOD2 were knocked down by siRNA, followed by CSE and SRT1720 treatment. Then, p62, LC3 I and LC3 II expression was determined. **E–F** LC3 (**E**) and lysosome (**F**) and LDs were stained to show lipophagy. **G** Cellular lipid content was evaluated by Nile Red staining. **H** Expression of collagen I, α-SMA, CPT1a and PPARα. *p < 0.05 versus control, ^#^*p* < 0.05 versus CSE, ^&^*p* < 0.05 versus CSE + SRT1720 (n = 3)

Results showed in CSE-treated cells, SRT1720 decreased mtNOX4 and elevated SOD2 (Fig. 5A), couple with declined mtROS (Fig. 5B). Furthermore, we knocked down SOD2 and found that SOD2 siRNA blocked the effect of SRT1720 on autophagy flux (Fig. 5C, D), lipophagy (Fig. 5E, F), lipid accumulation (Fig. 5G) and the expression of PPARα, CPT1a, collagen I and α-SMA (Fig. 5H). These results demonstrated that the effect of SIRT1 on lipid metabolism and LF activation was mediated by mitochondrial oxidative stress.

### Clearing mtROS or activating SIRT1 can prevent LF activation, increase PPARα and CPT1a expression of LF in vivo

**Fig. 6:**
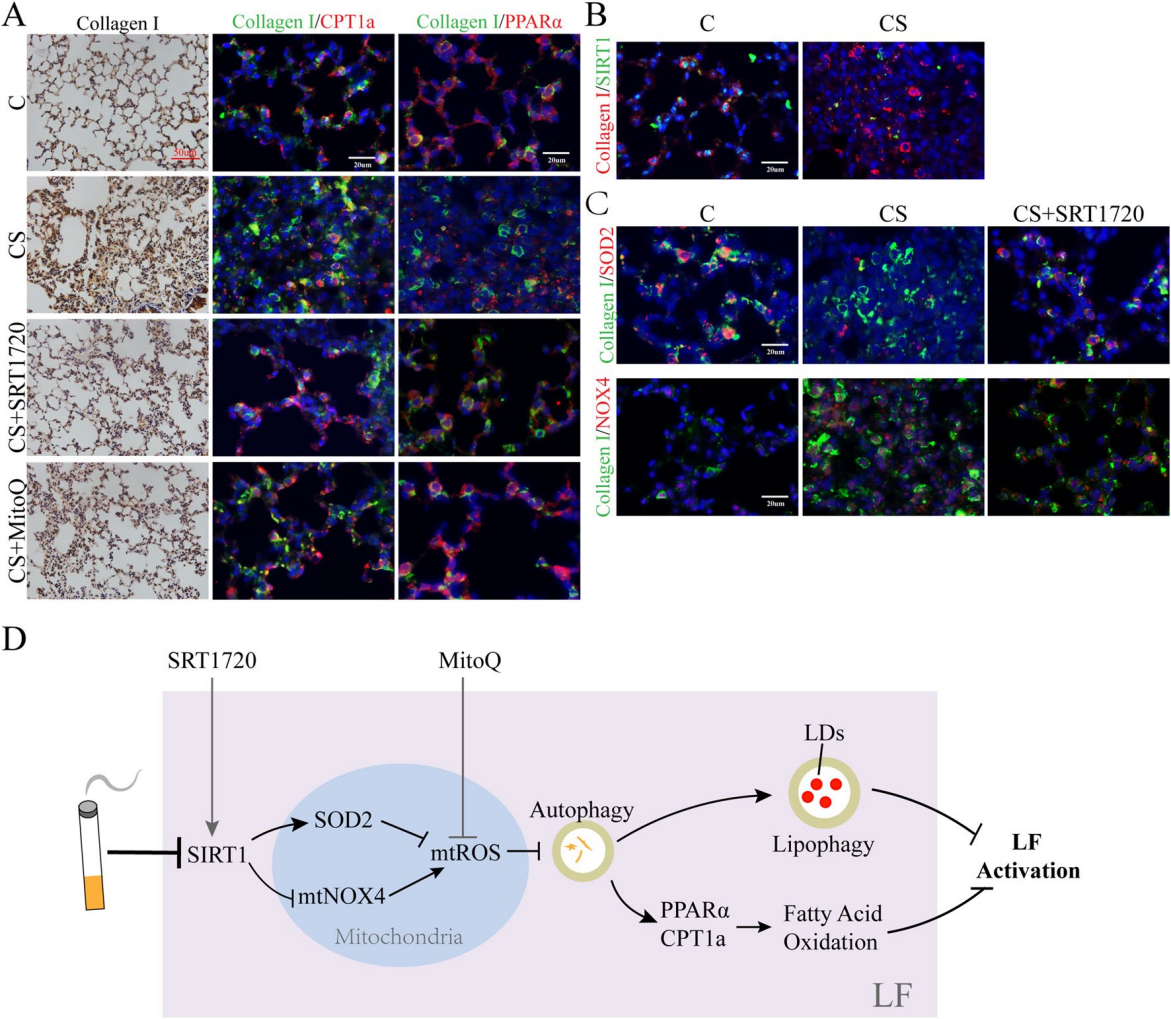
Targeting mtROS or SIRT1 regulated NOX4, SOD2, PPARα and CPT1a of LF in vivo. Mice exposed to CS were treated with MitoQ or SRT1720 at the start of smoking. 4 weeks later, lungs were harvested. **A**–**C** The expression of collagen I in lungs was examined by immunohistochemistry and CPT1a, PPARα, SIRT1, NOX4 and SOD2 level in LF (collagen I-positive cells) were detected by immunofluorescence. **D** CS decreased SIRT1 to activate LF by promoting mitochondrial oxidative stress, which dysregulated FAO and mitophagy through impairing autophagy flux. *CS* cigarette smoking

We previously revealed that MitoQ and SRT1720 can mitigate CS-induced pulmonary fibrosis [3]. Here we chose collagen I as the marker of LF based on two single-cell sequencing studies [23, 24] to further confirm the role of MitoQ and SRT1720 in mitochondrial oxidative stress and lipid metabolism of LF in smoking mice. And we found MitoQ decreased the level of collagen I (Fig. 6A) and increased the level of PPARα and CPT1a of LF (Fig. 6A).

Furthermore, we showed SIRT1 in LF of mice exposed to smoke was reduced (Fig. 6B). SRT1720 declined the level of collagen I in lungs (Fig. 6A). Moreover, in LF, NOX4 was downregulated by SRT1720 and SOD2, PPARα and CPT1a was increased (Fig. 6A, C). Therefore, clearing mtROS or targeting SIRT1 can regulate mitochondrial oxidative stress, FAO and protect against activation of LF in smoking mice.

## Discussion

In the present study, we centered on mtROS to explore how it was regulated by CS and how it contributed to LF activation. Our results showed that CS-induced mtROS was due to the imbalance of mtNOX4 and SOD2 caused by decreased SIRT1. And it activated LF by dysregulating PPARα/CPT1a-mediated FAO and lipophagy, both of which resulted from blocked autophagy flux (Fig. 6D).

mtROS, a critical player in IPF development and LF activation [25], can be induced by CS in a variety of cells [3, 26–28]. Our previously study has suggested mtROS may be a therapeutic target for CS-related pulmonary fibrosis [3]. However, how it worked in LF is incompletely known. In the present study, we explored how CS regulated mtROS and how mtROS participated in CS-induced LF activation. We previously proved increased NOX4 was a contributor of LF activation [4]. Studies reported that NOX4 can be localized in mitochondria and the elevation of mtNOX4 was related with LF activation [4]. Here we demonstrated CSE increased mtNOX4. And consistent with previous researches [27], we also evidenced the expression of SOD2, the main antioxidant enzyme of mitochondria, was reduced. Therefore, CSE disrupted mitochondrial redox balance. Furthermore, we first unveiled the inhibitory effect of MitoQ on LF activation. Similarly, studies also demonstrated MitoQ can prevent the activation of cardiac and nasal fibroblast [29, 30]. In addition, the antifibrotic effect of MitoQ has been evidenced in lung [3], liver [31] and kidney [32]. Moreover, the safety of MitoQ has been confirmed by Phase II clinical trials [33]. Altogether, MitoQ may be an effective and safety treatment for IPF or other smoking-related disease.

Lipid metabolism has been reported to be disrupted in fibrotic lungs [7, 34] and participate in fibroblast activation [35]. It can also be dysregulated by CS [9]. As lipid metabolism is closely modulated by mitochondria, we explored whether CS-induced mtROS activated LF by dysregulating lipid metabolism. Firstly, we examined the expression of CPT1a, since it is a key rate-limiting enzyme of mitochondrial FAO and is involved in kidney [10] and liver fibrosis [11]. And we found CSE downregulated CPT1a of LF. In addition, we proved that PPARα, the upstream regulator of CPT1a, was also inhibited. Consistent with previous study that PPARα activators exert anti-fibrotic effects in liver [12], kidney [13], heart [14] and lung [15], we revealed that PPARα activator prevented CSE-induced LF activation by elevating CPT1a. Furthermore, we proved that decreased FAO was due to mtROS. CPT1a inhibitor ETO as well as oleic acid, a fatty acid that is upregulated in the plasm of IPF patients [36] and has pro-fibrotic effect [37], can inhibit the anti-fibrotic effect of mitoQ, demonstrating that mtROS activated LF by dysregulating PPARα/CPT1a-related FAO. These results implying that pulmonary lipid metabolic state may have diagnosis potential for IPF. And interfering it may be effective for IPF.

To make clear how mtROS dysregulated lipid metabolism, we paid our attention to autophagy due to following reasons. Firstly, autophagy can prevent LF activation and fibrosis through multiple pathways [38, 39], but how it worked on lipid metabolism in CSE-treated LF is rarely explored. Secondly, autophagy is critical for mitochondrial homeostasis [40] which plays an important role in lipid metabolism. And our previous studies showed autophagy flux was impaired by CSE in an oxidative stress-related pathway [4]. Thirdly, compelling evidences indicated lipophagy, a process of autophagy-mediated LDs degradation, is necessary for lipid homeostasis [22]. Abnormal lipophagy has also been reported to be involved in fibrotic diseases [41]. Therefore, it is reasonable to postulate that CSE-induced mtROS disrupt lipid metabolism in an autophagy dependent pathway. As we expected, we found autophagy flux can be blocked by mtROS. And it played an essential role in lipid metabolism not only by regulating PPARα and CPT1a but also inhibiting lipophagy. In addition, our results indicated that mtROS did not influence the transfer of LDs to autophagosomes, but inhibited the transfer of LDs to lysosomes. This result was consistent with our previous finding that CS-induced dysfunction of lysosome contributed to impaired autophagy flux [4]. We supposed the effect of autophagy on PPARα/CPT1a-mediated FAO was associated with mitophagy, a process essential for mitochondrial homeostasis. However, the regulatory effect of lipophagy on FAO has also been reported [22]. Therefore, it still needs to be explored whether autophagy regulated FAO by mitophagy or lipophagy. The importance of autophagy in lipid metabolism was further confirmed by results that CSE-untreated LF has compensatory capacity for lipid homeostasis maintenance in the presence of ETO or OA. However, in BA-treated LF, the capacity was lost even in the absence of CSE. Thus, CSE-induced mtROS blocked autophagy flux to dysregulated lipid metabolism by inhibiting PPARα/CPT1a and lipophagy. The study further revealed the mechanism of autophagy in pulmonary fibrosis.

As we mentioned above, mtNOX4/SOD2-mediated mtROS played a critical role in CSE-induced LF activation. Hence, it is noteworthy to find the mechanism by which CSE disrupt the balance of mtNOX4 and SOD2. In the present study, we focused on SIRT1. For one thing, the negative effect of SIRT1 on mitochondrial oxidative stress has been confirmed in multiple organs, such as kidney [42], liver [43] and lung [3]. Moreover, it has been reported that NOX4 and SOD2 can be modulated by SIRT1 [16, 44]. For another, its anti-fibrotic effect has been confirmed in pulmonary fibrosis [18, 45]. And studies showed SIRT1 can protect against TGF-β-induced LF activation [18]. It also modulates lipid metabolism [19] and autophagy [3]. However, whether it can rebalance mtNOX4 and SOD2 and thereby regulate autophagy and lipid metabolism to protect against CSE-induced LF activation is uncertain. Here, we uncovered that SIRT1 rescued autophagy flux by rebalancing mtNOX4 and SOD2. And it promoted lipophagy and PPARα/CPT1a expression in an autophagy-dependent pathway. Therefore, activating SIRT1 may be a valuable treatment against pulmonary fibrosis or CS-related disorders. However, there are still some challenges, since changes of SIRT1 is complex. For example, CSE inhibited SIRT1 activity in alveolar epithelial type II cells [3] but decreased SIRT1 expression in LF. This reminded us that it would be more rational to perform different interventions in different cell types because of the complexity of the human body, although the anti-fibrotic effect of whole body SRT1720 stimulation has been confirmed in mice.

The utility of α-SMA as the marker of activated LF was challenged recent years as it was only upregulated in a subset of these cells [23]. Moreover, in lungs of control mice, α-SMA-positive LF is rarely detected, which make it difficult to compare the difference of indicators between control and pro-fibrotic LF in vivo. A present unbiased single-cell RNA sequencing study revealed that col1a1 is expressed in 99.8% activated LF, 80.4% nonactivated LF and 4.7% non-LF. Although not a perfect marker, it is better than others such as α-SMA, which is expressed in 63.6% activated LF, 11% nonactivated LF and 4.1% non-LF [23]. The superiority and rationality of col1a1 as the marker of LF was further confirmed by another single-cell RNA sequencing study [24]. So, we chose col1a1 as the marker of LF to detect the different expression of above indicator in LF in vivo.

## Conclusion

Taken together, we demonstrated CS-induced mtNOX4/SOD2 mediated mtROS contributed to LF activation by decreasing PPARα/CPT1a-mediated FAO and lipophagy, which resulted from blocked autophagy flux. The reduction of SIRT1 expression was responsible for CS-induced mitochondrial oxidative stress. Consequently, CS decreased SIRT1 to activate LF by promoting mitochondrial oxidative stress, which dysregulated lipid metabolism through impairing autophagy flux. Targeting these events may have therapeutic effect for pulmonary fibrosis.

## Supplementary Information

**Additional file 1 **Original blots of Western Blot analysis. The figure legend of this file is the same as the legend of the corresponding figure in the main text.

**Additional file 2 **MTT test for compounds used to treat cells. Cells were treated as indicated and their concentrations were showed in Materials and Methods section. Then, MTT tests were performed following manufacture’s instruction (Beyond, Shanghai, China). *CSE* cigarette smoke extract, *MitoQ* mitoquinone, *Feno* fenofibrate, *OA* oleic acid, *ETO* etomoxir, *BA* bafilomycin.

## Data Availability

All data generated or analyzed during this study are included in this published article.

## Abbreviations

BA: Bafilomycin
CS: Cigarette smoking
CSE: Cigarette smoke extract
ETO: Etomoxir
FA: Fatty acid
FAO: Fatty acid oxidation
Feno: Fenofibrate
IPF: Idiopathic pulmonary fibrosis
LDs: Lipid droplets
LF: Lung fibroblast
mtNOX4: Mitochondrial NADPH oxidase 4
mtROS: Mitochondrial reactive oxygen species
MitoQ: Mitoquinone
OA: Oleic acid
