# Supplementary figures and images for: SIRT1 prevents cigarette smoking-induced lung fibroblasts activation by regulating mitochondrial oxidative stress and lipid metabolism
Source: J Transl Med. 2022 May 14;20:222. doi: 10.1186/s12967-022-03408-5 (PMC9107262; doi:10.1186/s12967-022-03408-5)

Fig 1

C

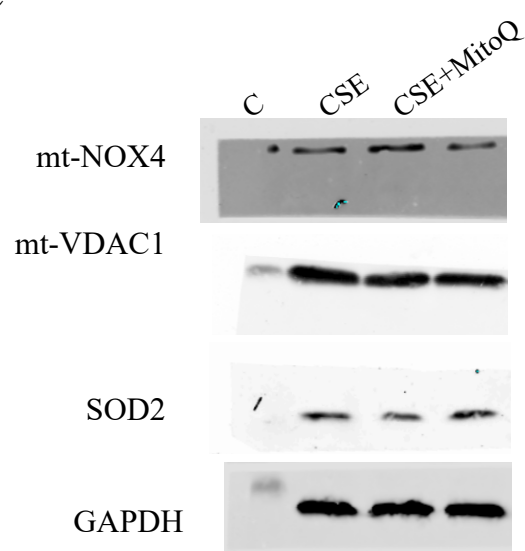

D

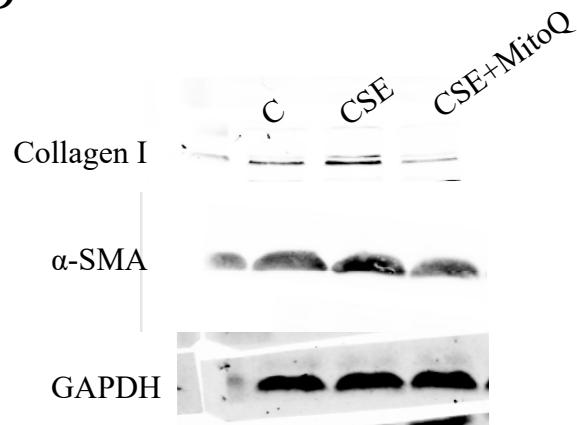

Fig 2

A

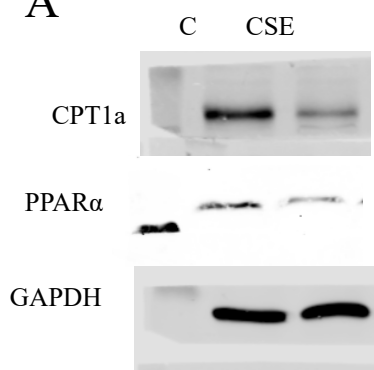

C

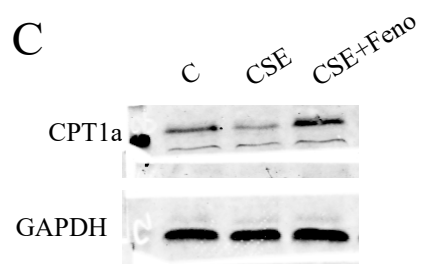

D

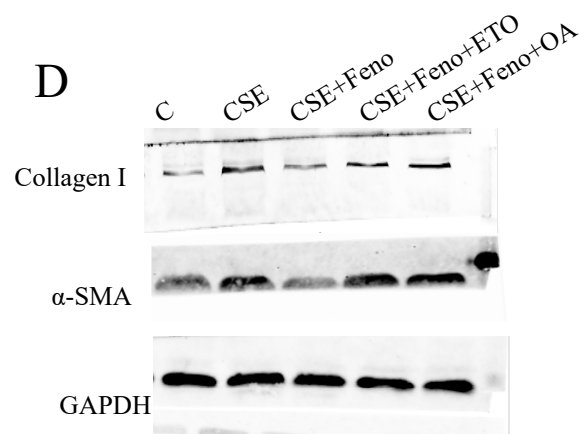

E

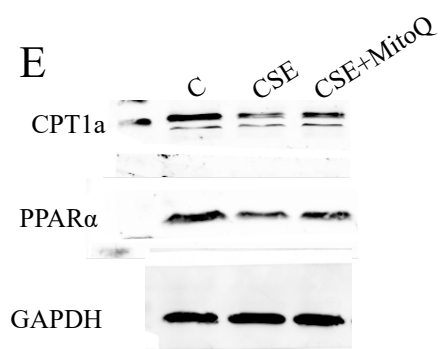

G

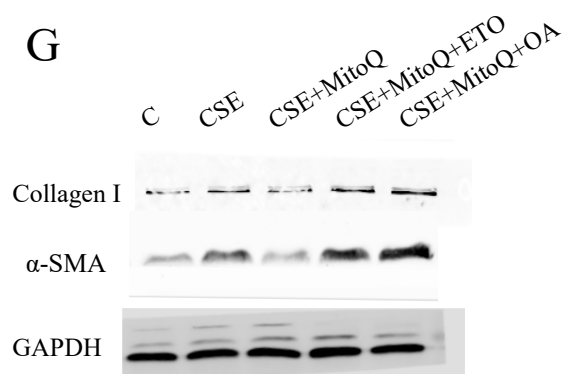

Fig 3

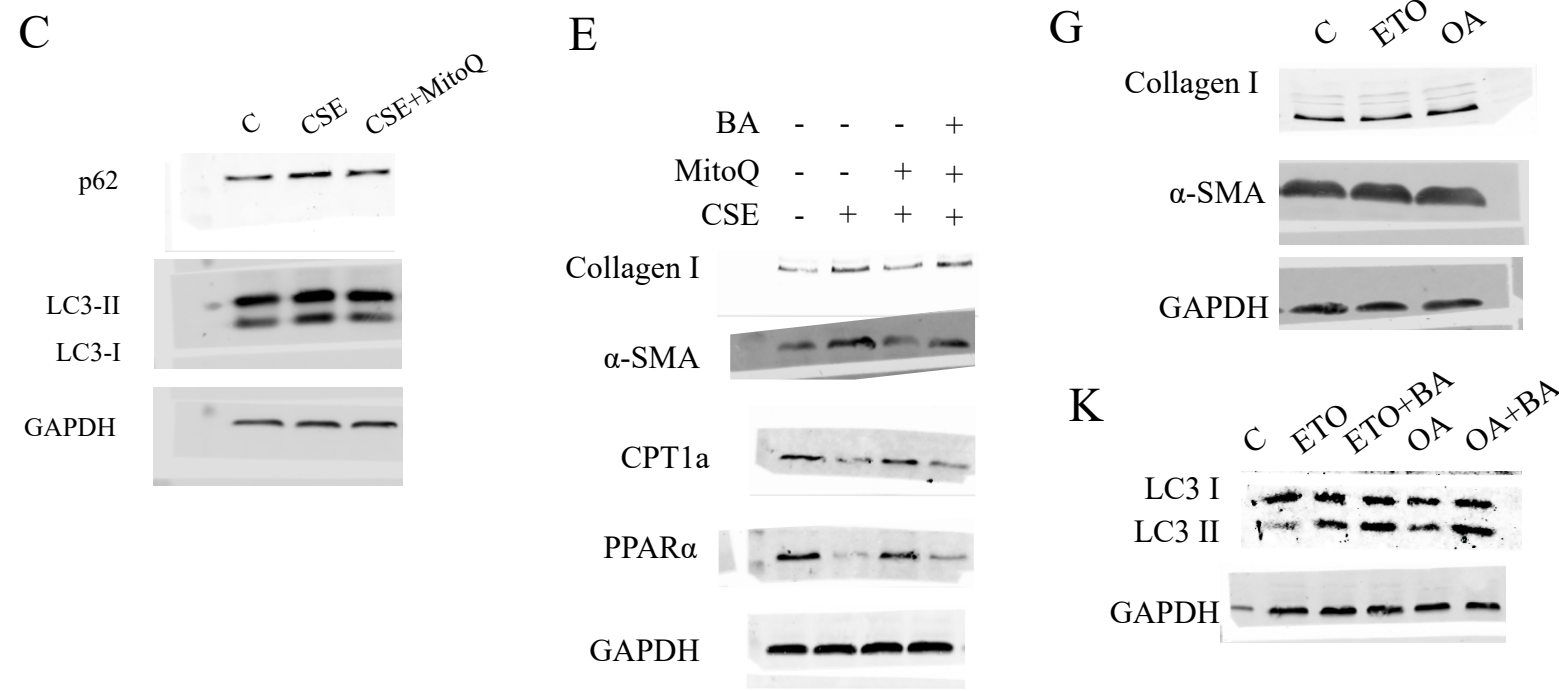

Fig 4

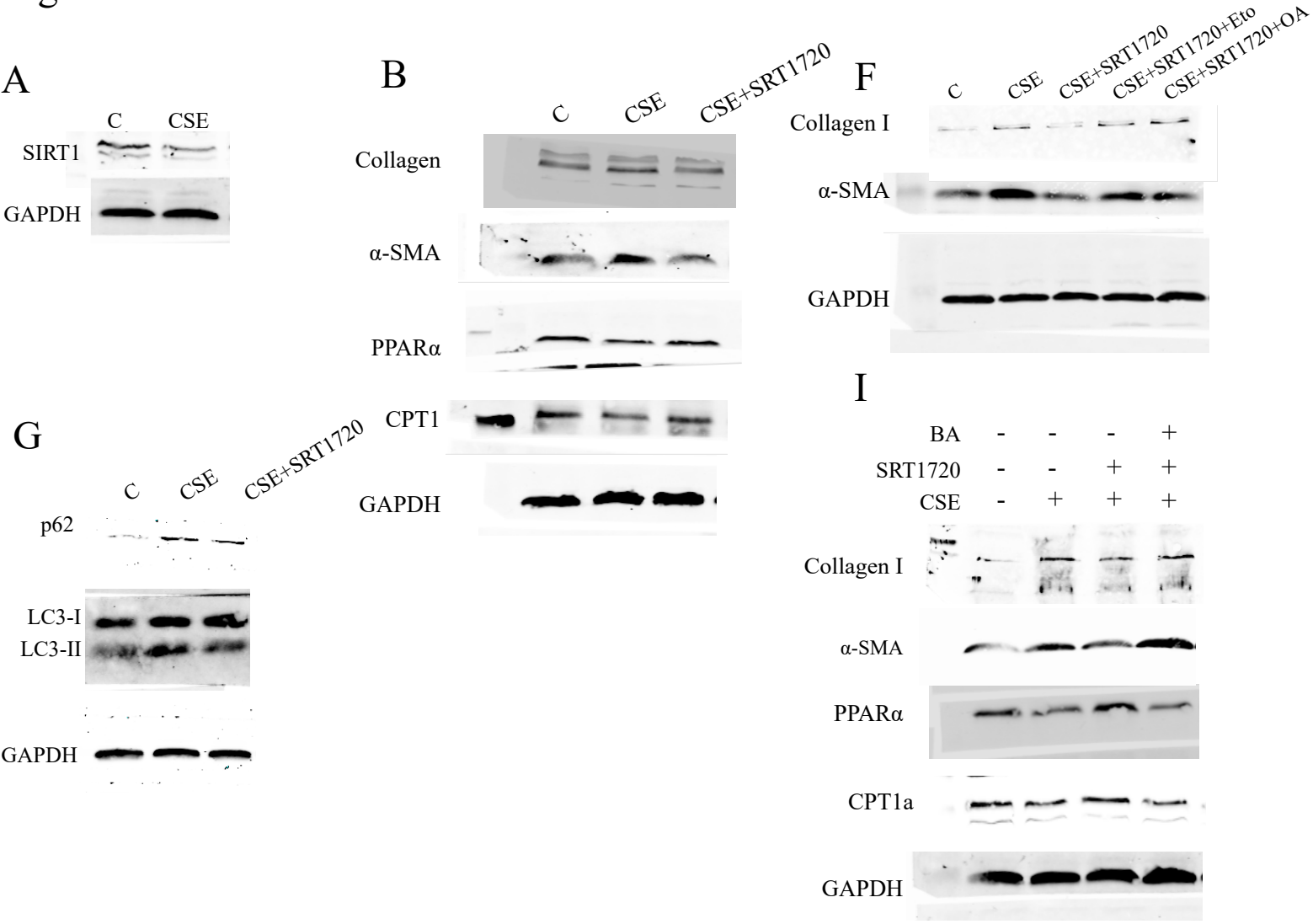

Fig5

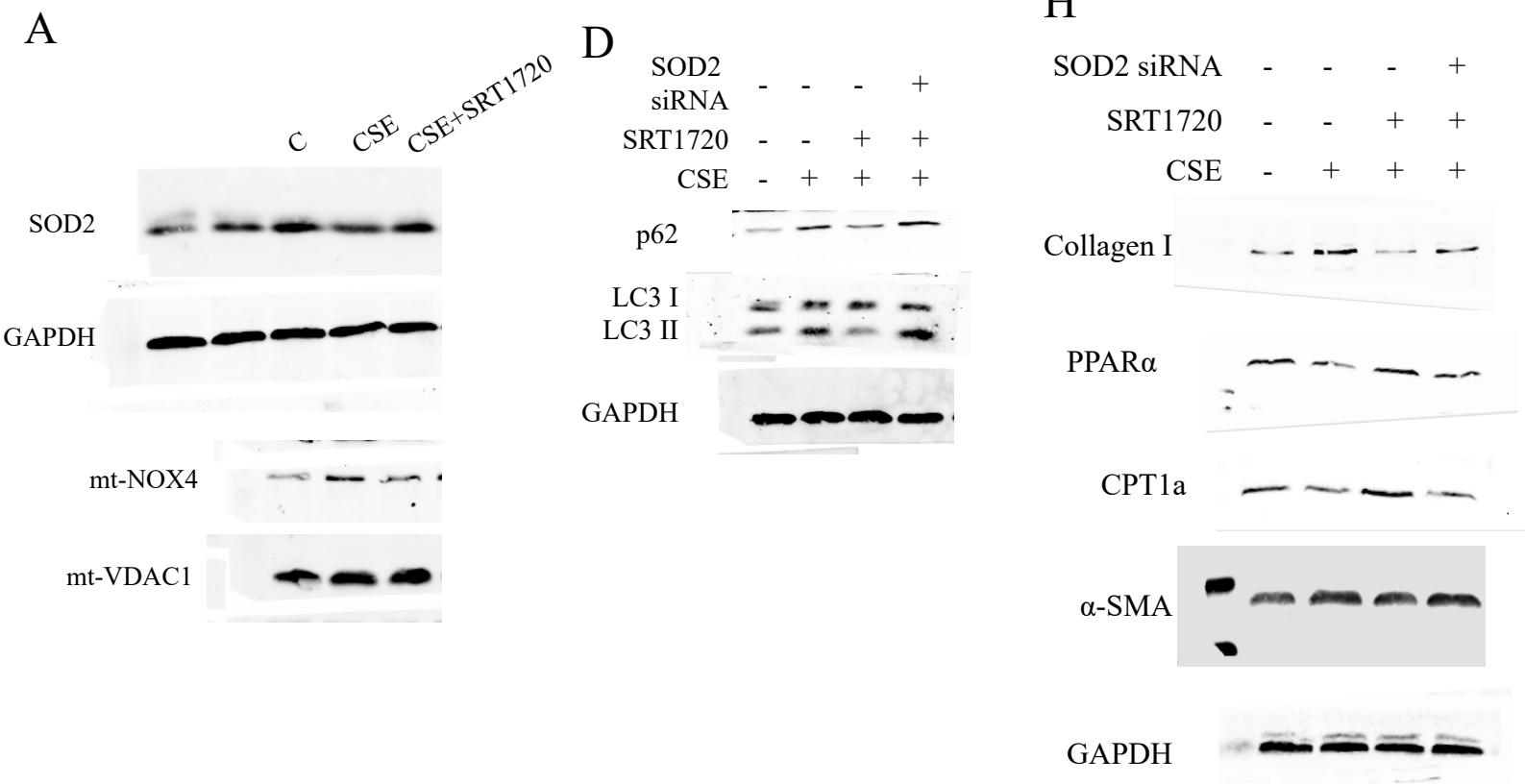

Supplement: Supplementary file 1 — Additional file 1 Original blots of Western Blot analysis. The figure legend of this file is the same as the legend of the corresponding figure in the main text. [file 12967_2022_3408_MOESM1_ESM.pdf]
