# Supplementary material for: SIRT1 prevents cigarette smoking-induced lung fibroblasts activation by regulating mitochondrial oxidative stress and lipid metabolism
Source: J Transl Med. 2022 May 14;20:222. doi: 10.1186/s12967-022-03408-5 (PMC9107262; doi:10.1186/s12967-022-03408-5)
Supplement: Supplementary file 2 — Additional file 2 MTT test for compounds used to treat cells. Cells were treated as indicated and their concentrations were showed in Materials and Methods section. Then, MTT tests were performed following manufacture’s instruction (Beyond, Shanghai, China). CSE cigarette smoke extract, MitoQ mitoquinone, Feno fenofibrate, OA oleic acid, ETO etomoxir, BA bafilomycin. [file 12967_2022_3408_MOESM2_ESM.pdf]

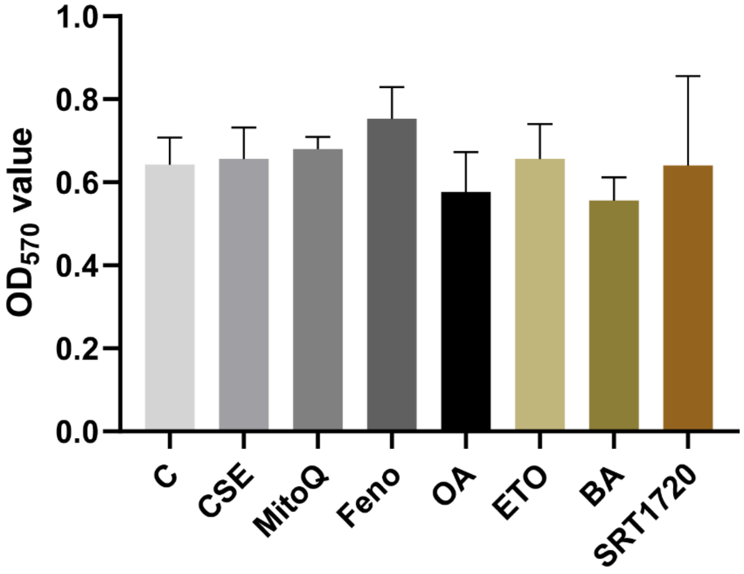

MTT test for compounds used to treat cells. Cells were treated as indicated and their concentrations were showed in Materials and Methods section. Then, MTT tests were performed following manufacture's instruction (Beyond, Shanghai, China). CSE, cigarette smoke extract; MitoQ, mitoquinone; Feno, fenofibrate; OA, oleic acid; ETO, etomoxir; BA, bafilomycin.
